# Spinal Cord Stimulator Explant and Revision Complicated by Syrinx Formation: A Case Report and Literature Review

**DOI:** 10.7759/cureus.5299

**Published:** 2019-08-01

**Authors:** Sameer S Ali, Ilya Bragin, Arjumond Y Khan, Hajime Tokuno, Pavan Tankha

**Affiliations:** 1 Neurology, Veterans Affairs Connecticut Healthcare System, West Haven, USA; 2 Neurology, St. Luke's University Health Network, Bethlehem, USA; 3 Pain Management, Veterans Affairs Connecticut Healthcare System, West Haven, USA

**Keywords:** spinal cord stimulation, spinal cord injuries, neuropathic pain, dorsal column stimulator, percutaneous leads, syrinx, spinal cord stimulator

## Abstract

Spinal cord stimulation (SCS) has been shown to be a safe, effective, and drug-free treatment option for many chronic pain conditions including refractory low back pain. The most commonly reported complication of SCS is equipment failure. We report a case of spinal cord injury (SCI) during SCS explant and revision.

This 61-year-old female veteran complained of intermittent shock-like sensations 3-4 times a week for three months prior to her clinic visit. The device was initially implanted in 2009 secondary to neurogenic claudication with appropriate relief. The battery was replaced in 2015. Pain Management Service referred the patient to neurosurgery for replacement of the original SCS unit. Immediately following surgery she complained of severe left lower extremity pain concentrated in the medial thigh radiating into the groin and buttock. She also complained of pain, weakness and numbness in both legs (left more than right). Magnetic resonance imaging (MRI) revealed an edematous area in the left spinal cord between T11-T12. The patient was placed on steroids, ketamine infusion for pain control, and MRI the next day showed slight improvement of the edema and she was discharged home. Follow-up MRI two months later revealed mild diminution in the size of the cord edema. Her pre-operative shock-like sensations had not returned.

While rare, spinal cord injury can occur and should be identified and managed expeditiously. Our case here reports for the first time an association between SCS explant/revision and syrinx formation.

## Introduction

Spinal cord stimulation (SCS) has been proven effective for multiple chronic pain syndromes with strong evidence [1-3]. SCS applications and technologies are rapidly advancing. High-level evidence exists for the safety, efficacy, and cost-effectiveness (Level I-II) of traditional SCS therapies in the treatment of chronic refractory low back with predominant limb pain. There is now Level I evidence for both dorsal root ganglion SCS and high-frequency SCS that demonstrates compelling results compared with traditional therapies. Overall, scientific literature demonstrates SCS to be a safe, effective, and drug-free treatment option for many chronic pain etiologies [4]. Our case involves the development of a previously unreported complication of syrinx formation resulting in spinal cord injury (SCI) causing a rare posttraumatic myelopathy.

## Case presentation

A 61-year-old female veteran with history of percutaneous lead SCS implant in 10/2009 for neurogenic claudication with good relief of symptoms with subsequent SCS revision with generator relocation due to discomfort in 8/2015 presented in 8/2017 to pain management for new symptoms. She complained of intermittent shock-like sensations traveling from her midline lower thoracic insertion incision region of the lead extending up the spine a few inches occurring multiple times a day, 3-4 days a week for previous three months. The sensation would occur positionally while leaning back. She denied any back injury and felt the SCS was providing relief for her chronic symptoms. Interrogation of the SCS was normal. She was referred to neurosurgery for explantation and replacement with a magnetic resonance imaging (MRI) compatible device with a goal of eliminating the shock-like sensations, continue appropriate pain coverage and allow for future MRI scans.

**Figure 1:**
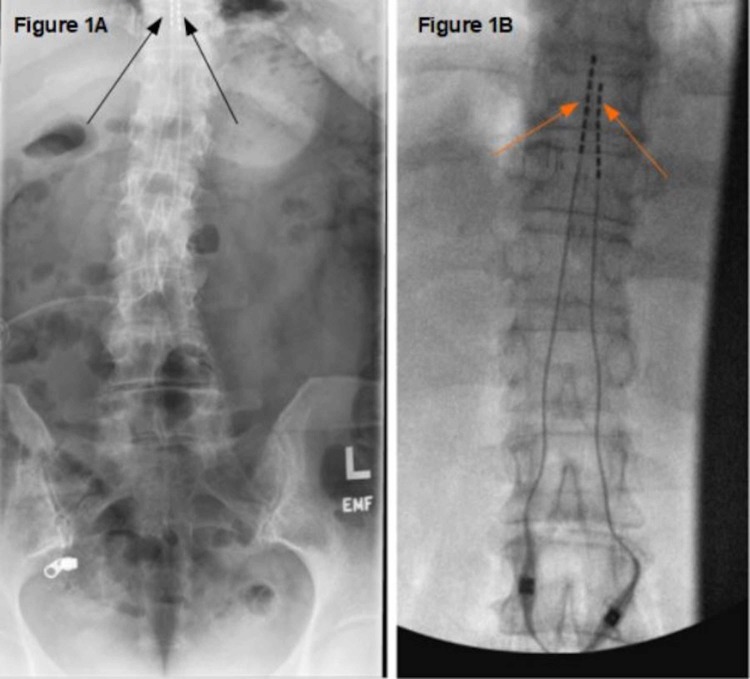
Preoperative and postoperative pictures depicting old and new spinal cord stimulator apparatus (A) Preoperative lumbar radiograph with two percutaneous leads at T9/10 (Black arrows). (B) Postoperative placement of two percutaneous leads at T8/T9 (Orange arrows).

Surgery was performed two months later via midline lower thoracic incision and right flank incision under monitored anesthesia care (MAC). Her previous two leads which were positioned at the T9/T10 level were both removed and two more epidural leads were placed percutaneously through the L1/L2 epidural space into the T8/T9 level (Figure 1A, 1B).

Upon waking from surgery, she complained of severe left lower extremity sharp, spasmodic pain concentrated in the medial thigh going into the groin and buttock. Additionally, shooting pains down the extremity, patchy numbness proximally becoming circumferential numbness below the left knee, and distal greater than proximal weakness with very minimal movement of toes all were suddenly present. There was no contralateral pain, numbness, or weakness.

**Figure 2:**
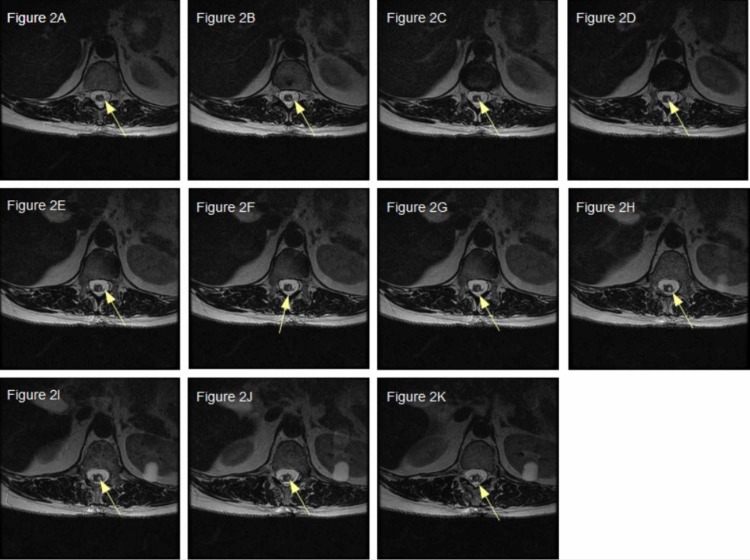
Iatrogenically-induced syrinx (A-K) Consecutive, rostral to caudal, axial T2-weighted MRI images with yellow arrows directed at T2 hyperintensity representing the syrinx between T11 and T12.

**Figure 3:**
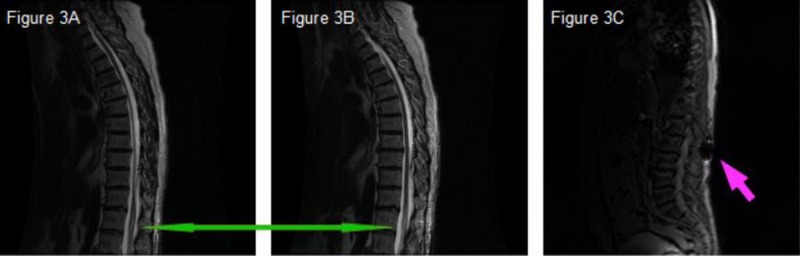
Syrinx and needle insertion during the explant/revision (A-B) Green arrow directed to syrinx on sagittal T2-weight MRI images. (C) Gradient echo sequence with pink arrow directed at needle insertion point at L2/L3 spinal interspace directed rostrally.

The anesthesia team cleared the patient of anesthesia complications. An urgent non-contrast MRI of the thoracic spine showed a syrinx in the left hemicord at the level of T11 extending to T12 appearing to be posttraumatic as there was a focus of increased T2 signal with a small amount of blood (Figure 2A-2K; Figure 3A-3C).

The patient was immediately started on dexamethasone, gabapentin, opioids, diazepam and ketamine infusion for analgesia and admitted to the neurosurgical inpatient service.

Repeat MRI scan postoperative day 3 showed slight decrease in cord edema and smaller diameter of the longitudinal spread of the T2 intramedullary signal change correlating with slight improvement of symptoms. Her strength and distal numbness showed some improvement, groin pain resolved, however she noticed medial left knee pain associated with bladder fullness but with no difficulty voiding. She was taken off ketamine at this point and steroids started to be weaned. She was able to ambulate with walker and start physical therapy. Additionally, her SCS was reprogrammed with the added benefit of covering her pre-existing bilateral lower extremity pain. The intermittent shock sensations did not return after surgery. She was discharged to a rehabilitation facility and had a three-week post-operative follow-up visit with neurosurgery. Given her left lower extremity symptoms potentially exacerbated by the SCS, the SCS was reprogrammed to cover only her right lower extremity (RLE). She had her outpatient pain management clinic follow-up two months post-operatively and was frustrated that although she regained some strength, she was still having difficulty at work as she is a bus driver. She also felt that the pain level hadn’t improved and numbness remained the same. Repeat MRI of the thoracic spine without contrast on this day showed mild diminishment of syrinx size.

## Discussion

Over the past 40 years of use, the complication rates of SCS have been well defined in the literature. The most common reported complication is equipment failure without neurologic injury. There is a significant rate of minor complications, many of which require further surgical intervention to manage, including lead migration or implant infection, although such complications do not directly threaten patient life or function [5].

Interestingly the incidence of one of the most devastating complications, SCI, remains largely unknown. This includes after SCS trial, implantation, and revision. It is considered to be a rare complication and no one has described a syrinx in the setting of SCS [6]. The syrinx itself was not longitudinally extensive, spanning a spinal level, but did have clinically significant associated symptoms. The procedure itself, including a potentially puncture with a small gauge needle or the stimulator leads themselves, may have led to this complication. Interestingly, despite the development of the syrinx, there was no cerebrospinal fluid leak radiographically, or a low-pressure headache, clinically to suggest dural puncture. Importantly, pre-procedural MRI did not demonstrate syrinx pointing to the procedure as directly contributing to it.

One retrospective study reviewed the percutaneous versus paddle insertion SCS techniques to quantify the incidence of SCI. This study showed that the overall incidence of SCI in SCS is low (2.13%), supporting that SCS is a safe procedure. No significant difference was found in the rates of SCI or spinal hematoma between the percutaneous and paddle groups [1]. There are a handful of case reports in literature mentioning various complications. There is one case where a patient developed a cervical spinal cord injury after a fall with the electrodes of the spinal cord stimulator working as a space occupying mass and inducing the injury [7]. The puncture of the dura during electrode placement while performing SCS revision led to quadriparesis in one patient where even after successful removal of the electrode the patient continued to neurologically decline [8]. Additionally, there have been limited reports of neurologic injury secondary to infection, cord contusion, actual needle penetration of the spinal cord, epidural hematoma and cord compression from epidural hematoma [6]. There is also a reported case of cord compression from epidural fibrosis associated with percutaneously placed spinal cord stimulation electrodes [9]. There is a case report demonstrating thoracic SCS lead fibrosis causing permanent paraplegia [10]. Epidural hematoma reports have been described in context of both the removal of percutaneous spinal cord stimulator leads and also in a case with spinal cord stimulator trial lead placement in someone taking aspirin [11, 12]. The incidence of epidural hematoma is low and according to one retrospective study, no cases of epidural hematoma following percutaneous SCS lead placement were identified including over 100 patients taking aspirin or NSAIDs [2, 13-15].

## Conclusions

While rare, spinal cord injury can occur and should be identified and managed expeditiously. Our case here reports for the first time an association between SCS explant/revision and syrinx formation. The spinal cord injury resulting from this syrinx formation has led to a post traumatic myelopathy. Thankfully our patient has had slow but steady clinical improvement. Overall SCS is considered to be a safe procedure however further insight into the true incidence of neurologic sequelae is warranted and syrinx formation is another potential complication that can occur that has not been previously reported in this context.
